# Utility and reliability of the Clinical Frailty Scale in patients scheduled for major vascular surgery: a prospective, observational, multicentre observer-blinded study

**DOI:** 10.1186/s13741-022-00240-9

**Published:** 2022-01-31

**Authors:** Reema Ayyash, Joanne Knight, Elke Kothmann, Mohamed Eid, Katie Ayyash, Kerry Colling, David Yates, Aileen Mill, Gerard Danjoux

**Affiliations:** 1 South Tees Hospitals NHS Foundation Trust, Middlesbrough, UK; 2 Gateshead Health NHS Foundation Trust, Gateshead, UK; 3 The University Hospital of North Tees NHS Foundation Trust, Stockton-on-Tees, UK; 4 York & Scarborough Teaching Hospitals NHS Foundation Trust, York, UK; 5 Vertebrate Biodiversity and Ecology, Newcastle University, Newcastle, UK; 6 Hull York Medical School, York, UK; 7 School of Health and Life Sciences, Teesside University, Middlesbrough, UK; 8 North Yorkshire Academic Alliance of Perioperative Medicine, York, UK

**Keywords:** Frailty, vascular surgical procedures, pre-operative care, reproducibility of results, Edmonton Frailty Scale, Clinical Frailty Scale

## Abstract

**Background:**

Frailty is a distinctive health state associated with a loss of physiological reserve that results in higher rates of perioperative complications and impaired return to pre-morbid functional status. It is prevalent in the vascular population; however routine assessment is not common despite national guidance to the contrary. We aimed to evaluate the reliability of the Clinical Frailty Scale in assessing frailty in the surgical vascular population.

**Methods:**

In this prospective, observational, observer-blinded study, we compared assessment of frailty in patients scheduled for major vascular surgery attending the pre-operative assessment clinic using the Clinical Frailty Scale against the Edmonton Frailty Scale.

The study investigator completed the Edmonton Frailty Scale assessment; this was compared to the Clinical Frailty Scale assessments performed by the pre-assessment consultant and pre-assessment nurse, who were blinded to the Edmonton Frailty Scale score. The inter-rater reliability of the Clinical Frailty Scale between the pre-assessment consultant and pre-assessment nurse was determined by comparing their frailty scores for each patient.

**Results:**

Ninety-seven patients were included in the analysis (median age 72 years, 84% male and 16% female). There was a moderate level of agreement between the Edmonton and Clinical Frailty Scale score for both consultants (87.6% agreement) and pre-assessment nurses (87.6% agreement). There was a substantial level of agreement between consultants and pre-assessment nurses for the Clinical Frailty Scale (89.7% agreement)

**Conclusions:**

The Clinical Frailty Scale is a useful tool to assess frailty in the vascular surgical population. It is more practical than the Edmonton Frailty Scale: quick to complete, requires minimal training and can be used when physical disability is present.

**Trial registration:**

The study was approved by the Wales Health and Care Research Ethics Service (REC reference 17/WA/0160, IRAS 201173). Trial registration: NCT03403673. Registered 19 January 2018, https://clinicaltrials.gov/ct2/show/NCT03403673

## Background

Frailty is a distinctive health state associated with, but not causally related to, the ageing process. While difficult to define, researchers often use Fried’s five frailty characteristics: sedentary behaviour, poor grip strength, decreased gait speed, unintentional weight loss and low energy levels (Fried et al., 2001). Patients with three or more features are considered frail, whilst pre-frailty is the stage within the frailty continuum whereby one or two of the Fried’s criteria are met (representing an increased risk of becoming frail) (Han et al., 2019). Individuals who are pre-frail or frail gradually lose their in-built physiological reserve, leaving them vulnerable to acute changes in health status triggered by events such as an infection or surgery, resulting in functional decline and prolonged recovery.

Compelling evidence from studies and meta-analyses has implicated frailty as an independent prognostic indicator for adverse perioperative outcomes and prolonged length of hospital stay, institutionalisation after discharge and higher 30- and 90-day mortality rates (Clegg et al., 2013; Song et al., 2010; Robinson et al., 2013). In addition, a recent study has associated frailty with a worsening in disability score following surgery not seen in non-frail patients (McIsaac et al., 2020). The pre-frail patient is at higher risk of perioperative complications compared to the non-frail patient (Han et al., 2019).

This is important given the substantial increase in the number of older people undergoing surgery in the last decade: in England there were 1.5 million surgical patients aged over 75 in 2006–2007 increasing to 2.5 million in 2014–2015 (Health and social care information centre, 2006-2007; Health and social care informaion centre., 2015). As a result, more old, frail patients with multiple co-morbidities present for surgery. In the major vascular surgical population frailty may be identified in approximately 50% of patients where robust screening efforts are implemented (Hewitt et al., 2015). Due to the high-risk nature of vascular surgery, frail and pre-frail patients undergoing such procedures appear to be at particularly high risk of adverse perioperative outcome and reduced survival in this setting (Clegg et al., 2013; Health and social care information centre, 2006-2007; Partridge et al., 2015; Wang et al., 2018; Donald et al., 2018).

Identifying pre-frailty and frailty pre-operatively is crucial to direct timely clinical shared decision-making with patients, facilitate risk factor modification, resource planning and optimisation of health outcomes (Amrock & Deiner, 2014). The Centre for Perioperative Care, including The Royal College of Anaesthetists and British Geriatric Society (Centre for Perioperative Care and British Geriatric Society, 2021) have set out national recommendations to assess frailty pre-operatively. Despite this, routine screening is lacking (Partridge, 2014). Several factors have been implicated: time constraints in highly pressured pre-operative clinics, gaps in training and education amongst healthcare professionals, lack of infrastructure and a lack of consensus on the most effective tool for use in the pre-operative setting (over 20 tools are available to researchers (Han et al., 2019)). Potential suitable and simple tools for use pre-operatively are the Edmonton Frailty Scale (EFS) (Rolfson, 2006) and Clinical Frailty Scale (CFS) (Rockwood, 2005).

The EFS has the greatest evidence base for use in this clinical setting. It incorporates 10 domains of frailty including cognitive impairment, balance and mobility. From the 10 domains a final score is calculated from 0 (non-frail) to 17 (extremely frail). The EFS is validated for use by non-geriatricians and a broad spectrum of other health care professionals with no prior medical training, without detriment to its reliability (Partridge et al., 2015; Rolfson, 2006 Dasgupta, 2009; Schmucker et al., 2019; Amabili et al., 2019). Several studies have shown an association between higher EFS scores and adverse outcomes including increased perioperative complications and prolonged hospitalisation (Partridge et al., 2015; Wang et al., 2018; Donald et al., 2018; Amrock & Deiner, 2014).

Other simpler tools such as the ‘Initial Clinical Impression’, an eyeball assessment made by a clinician within the first few minutes of a patient encounter about their suitability for surgery have also shown utility in this setting (O'Neill, 2016). Our previous research has demonstrated a > two-fold increase in medium-term all-cause mortality in patients presenting for assessment for major vascular surgery and being deemed unsuitable on Initial Clinical Impression. This increased risk of death was consistent for patients undergoing surgery and those receiving non-operative management (O'Neill, 2016). The CFS, developed at Dalhousie University as a means of summarising a multidimensional frailty assessment, is more detailed than the binary yes/no results provided by the Initial Clinical Impression. Initially, a seven-point pictorial scale [with descriptive anchors to assess increasing levels of frailty and dependency (Rockwood, 2005). It has since been revised to a nine-point pictorial scale to identify those living with severe and very severe frailty as well as those with a terminal illness (Rockwood, 2020). A score of one represents good physical health, with scores from 5 to 9 representing increasing levels of frailty from mild to very severe. The CFS lacks the depth and dimension assessed by the EFS, which is often seen as the standard; however, it may represent a pragmatic tool that is applicable, feasible and quick in a time pressured pre-assessment clinic. Importantly, the CFS has also demonstrated promising utility in predicting adverse perioperative outcomes from emergency general surgery, with a score of ≥ 5 recognised as the threshold for increased risk (Hewitt, 2019).

The aim of this prospective observational study was to evaluate the reliability of the CFS in assessing frailty in the vascular surgical population. The primary outcomes were to assess the utility of the CFS (the research tool) in capturing frailty as identified by the EFS (the reference tool) in patients presenting for major vascular surgery. The study also assessed the inter-rater reliability of CFS results between pre-assessment consultant and pre-assessment nurse. Secondary outcome measures included clinical outcomes and resource utilisation.

## Methods

This was a multicentre, prospective, observer blinded, observational study. The study was conducted across two NHS teaching hospitals: South Tees Hospitals NHS Foundation Trust (STHNFT) and The York Hospital (TYH).

Written informed consent was obtained from all patients prior to recruitment into the study. All vascular patients attending the pre-operative assessment clinic were screened for eligibility and provided with a patient information sheet. Patients ≥ 60 years of age scheduled for an elective major vascular surgical procedure were eligible for inclusion in the study. Patients were excluded if they were < 60 years of age, scheduled for a day case or non-arterial vascular surgical procedure, or if the patient declined or was unable to provide informed consent. Patient recruitment across the two centres was conducted between April 2017 and December 2018.

All frailty assessments were undertaken during the pre-operative assessment clinic visit. The CFS was completed independently for all patients using the comprehensive medical and social information collected during the pre-assessment for high-risk surgery consultation by a consultant anaesthetist and a staff nurse who were blinded to each other’s evaluations. One of the six research investigators across the two sites, blinded to the results of the CFS evaluations, performed the EFS at any point during the clinic visit. The consultant and pre-assessment nurse were also blinded to the results of the EFS.

Information relating to the secondary outcome measures was obtained following review of physical or computerised medical records. This included surgical procedure, postoperative morbidity using the Clavien-Dindo grading of complications, discharge destination, 30-day readmission, in-hospital mortality, length of critical care admission and length of hospital stay.

For the purpose of the analysis the two frailty scales measured were reduced to an ordinal classification: non-frail (EFS 0–5 and CFS 1–3); pre-frail (EFS 6–7 and CFS 4); and frail (EFS 8–17 and CFS 5–9). At the time of analysis, the CFS score of 4 had the descriptor title ‘vulnerable’ (now titled as ‘living with very mild frailty’) (Rockwood, 2020), similarly the EFS scores of 6–7 are classified as vulnerable (Dent, 2016). We used these vulnerable categories, implying patients are at risk of frailty, to be our pre-frail category.

The sample size for a sufficiently powered study (1–β = 0.8) was estimated using the method of Kraemer and Thiemann (1987) (Kramer, 1987). The effect size was 5 for the EFS and 3 for the CFS, sufficient to cause a patient to be assessed as the next-most severe category on the respective frailty scales. An unequal cohort of 75% non-frail and 25% frail was assumed. This yielded sample size estimates of 82 for EPS and 70 for CFS. The recruitment of approximately 100 patients therefore facilitated comparison of the ordinal categories of frailty but was not powered for identifyingstatistical significance of secondary outcome measures.

Frailty assessments derived from the CFS were compared to the assessment derived from the EFS for both the pre-assessment consultant and staff nurse independently. Levels of agreement and inter-rater reliability between EFS and CFS assessments were calculated with a percentage agreement and Cohen’s Kappa coefficient. Levels of agreement using the Kappa coefficient values were set at < 0 = no agreement; 0–0.20 = slight agreement; 0.21–0.40 = fair agreement; 0.41–0.60 = moderate agreement; 0.61-0.80 = substantial agreement; 0.81–1.0 = almost perfect agreement (Landis, 1977).

We calculated descriptive statistics for patient demographics, co-morbidities and procedural overview. Median and inter-quartile range (IQR) and standard deviation (SD) are reported for continuous variables and percentages for categorical variables.

## Results

**Fig. 1:**
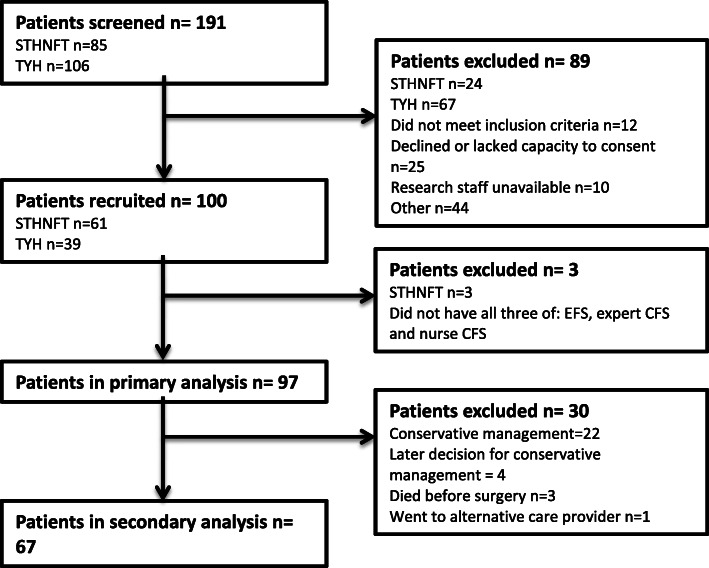
Participant flow through the study. STHNFT—South Tees Hospitals NHS Foundation Trust. TYH—The York Hospital

One hundred patients were recruited from a total of 191 screened. Figure 1 shows full details of patient flow through the study. Three patients were excluded, leaving 97 included in the primary analysis. The characteristics of this cohort are shown in Table 1. Sixty-seven patients progressed to surgery with type of procedure performed shown in Table 1.

**Table 1:** Characteristics, co-morbidities and surgical procedures

**Patient characteristics** ***n*** **= 97**	
**Age (median, (IQR))**	72 (60–92)
**Gender (number (%))**	
Male	81 (84%)
Female	16 (16%)
**Weight (kg) (median, (IQR))**	85 (34–134)
**Body mass index (kg/m**^**2**^**) (median, (IQR))**	29 (15–38)
**Height (cm) (median, (IQR))**	173 (150–193)
**Co-morbidities (*n*, % proportion)**	
Ischaemic heart disease	27 (28%)
Previous myocardial infarction	16 (16%)
Peripheral vascular disease	13 (13%)
Diabetes	18 (19%)
Hypertension	64 (66%)
Chronic obstructive pulmonary disease	18 (19%)
Chronic kidney disease	15 (15%)
Cerebrovascular disease	8 (8%)
Asthma	3 (3%)
**Surgical procedures (*n*, % proportion)** ***n*** **= 67**	
Open abdominal aortic aneurysm repair	32 (48%)
Endovascular abdominal aortic aneurysm repair	30 (45%)
Other surgery (aorto-bifemal graft, fem-fem crossover graft, femoro-popliteal bypass graft, internal iliac artery repair)	5 (7%)

Values are the median and inter-quartile range (IQR) for age, weight, height and BMI. Gender, co-morbidities and surgical procedure are displayed as a number and percentage proportion

**Fig. 2:**
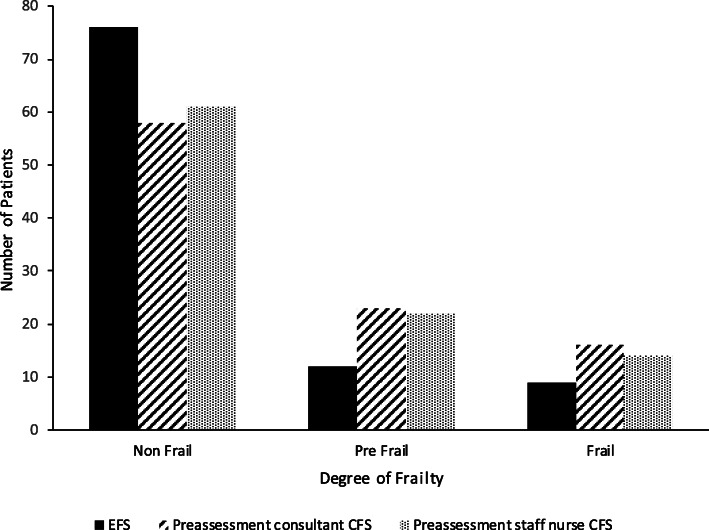
Frailty classification, using the Edmonton Frailty Scale (EFS) and Clinical Frailty Scale (CFS), of the patients used in primary analysis (*n* = 97)

Seventy-six of the 97 patients (78%) with full data sets for primary analysis were categorised as non-frail by the reference tool (EFS) (Fig. 2). The overall breakdown of numbers of frail, pre-frail and non-frail patients according to the EFS and CFS is shown in Fig. 2.

Six researchers conducted the EFS across the 2 trusts, 3 at STHNFT and 3 at TYH. 12 Consultants (3 at STHNFT and 9 at TYH) and 9 pre-assessment staff nurses (3 at STHNFT and 6 at TYH) were involved in completing CFS frailty evaluations across the 2 sites. Overall there was moderate agreement between clinician estimated CFS (both Consultant and staff nurse) and researcher EFS (Kappa coefficient 0.53 and 0.50 respectively) (Table 2). The level of agreement was slightly higher for Consultant CFS evaluations against EFS as opposed to staff nurse evaluation. Given this slightly higher level of agreement, the consultant CFS score was utilised for analysis of secondary outcome measures.

**Table 2:** Percentage agreement and Kappa co-efficient of frailty assessments

Paired background	Percentage agreement P(a)	Kappa statistics (k)	*Z*	*P* value
Researcher EFS and consultant CFS	87.6%	0.53	0.52	< 0.001
Researcher EFS and nurse CFS	87.6%	0.50	4.92	< 0.001
Consultant CFS and nurse CFS	89.7%	0.61	5.99	< 0.001

*EFS* Researcher Edmonton Frailty Scale assessment compared to the pre-assessment consultant, *CFS* Clinical Frailty Scale assessment; researcher Edmonton Frailty Scale assessment compared to staff nurse Clinical Frailty Scale assessment: and consultant Clinical Frailty Scale assessment compared to Staff Nurse Clinical Frailty Scale assessment across both hospital sites. Levels of agreement using the Kappa coefficient values were set at < 0 = no agreement; 0–0.20 =slight agreement; 0.21–0.40 = fair agreement; 0.41–0.60 = moderate agreement; 0.61–0.80 = substantial agreement; 0.81–1.0 = almost perfect agreement

The inter-rater reliability of CFS frailty scores between consultant and staff nurse demonstrated a substantial agreement (Kappa co-efficient 0.61) (Table 2).

A clinical decision to proceed with surgery was made for 75 patients. Of this group, three died prior to surgery (all non-frail), one chose to go to an alternative care provider and on review it was decided four patients should have conservative care (one non frail, one pre-frail and two frail). All these patients were subsequently excluded from secondary analysis. Of the remaining 67 patients proceeding to surgery, procedure by frailty group is shown in Table 3.

**Table 3:** Secondary outcome measures against patient frailty status

Secondary outcome measur		Non-frail (*n* = 49)	Pre-frail (*n* = 15)	Frail (*n* = 3)
Procedure	Open AAA	29 (59.2%)	3 (20.0%)	0
	Bypass graft	2 (4.1%)	3 (20.0%)	0
	EVAR	18 (36.7%)	9 (60.0%)	3 (100%)
Postoperative critical care admission (*n*, % proportion)		40 (81.6%)	11 (73.3%)	2 (66.7%)
Length of stay (mean, (SD))	Critical care	2 (2.41)	5 (8.07)	1 (0.07)
	Hospital	7 (4.84)	7 (6.24)	3 (0.58)
In-hospital mortality (*n*, % proportion)		0 (0%)	2 (13.3%)	0
30-day readmission (*n*, % proportion)		5 (7.9%)	0	0
Major morbidity: Clavien-Dindo Score ≥ 3A-5 (*n*, % proportion)		10 (20.4%)*	2 (13.3%)	0

Surgical procedure, number of critical care admissions, discharges home, in-hospital mortality and 30-day readmission (values as numbers and proportions of their frailty subgroup). The total length of critical care and hospital length of stay. Values are the mean and standard deviation [SD]

*Clavien-Dindo morbidity scores were unavailable for two patients in the non-frail group

Two patients died in-hospital following surgery (in-hospital mortality 3%), both of whom were categorised as pre-frail. Twelve patients suffered major morbidity (17.9%). A full breakdown of perioperative outcomes by frailty category is shown in Table 3. The correlation between frailty category and perioperative outcome was not assessed due to the imbalance of patients in each category.

## Discussion

This study, to the best of our knowledge, is the first to evaluate the reliability of the CFS against EFS in a pre-operative patient population. We have demonstrated several important findings. A moderate overall level of agreement in prediction of frailty status between CFS and EFS evaluations was demonstrated. There was very little difference observed between medical and nursing staff CFS evaluations, and their agreement with the researcher EFS evaluation, although the correlation was slightly higher for medical staff. Another finding was the substantial agreement demonstrated between medical and nursing colleagues in consistently assessing patient frailty status using CFS. This consistency in delivery across different healthcare professional (HCP) groups is possibly the more important finding given the range of HCPs involved in patient evaluations in the pre-operative setting. These findings together suggest that the CFS has good utility in the prediction of patient status across the frailty spectrum within a vascular population and the confines of this study.

The EFS has been documented to be a useful tool for stratification of fragility levels in routine pre-operative assessment screening. The EFS takes five minutes to complete with a trained professional and requires the patient to undertake a functional assessment, which may not be suitable for some vascular patients with functional disabilities or those who are very frail who may struggle with functional assessment domains of this assessment tool. The CFS, utilising information collected as part of a routine pre-operative assessment, expands on Initial Clinical Impression by evaluating specific domains of frailty including comorbidity, function and cognition. It takes seconds to undertake and is easy to grasp for newcomers to the concept of frailty. A recent study in the critical care setting highlighted that no particular training to use the CFS is necessary since description combined with illustrations is intuitive (Flatten, 2017). Similarly in our study, the pre-assessment consultant and nurse received no formal training prior to assessing frailty using the CFS. The descriptors and pictographs on the CFS assessment tool combined with the clinical consultation assessment were deemed sufficient to make a formal judgement about frailty status of the individual.

Critical care studies have also demonstrated good levels of agreement between CFS frailty scores performed by variously paired HCP combinations; medical student and critical care doctor (Pugh, 2017) or research co-ordinators, occupational therapists and geriatricians (Shears, 2018). In keeping with these studies we also observed that consultants rated a slightly greater proportion of patients to be frail compared to pre-assessment staff nurses.

In addition, our results confirm findings from a study in the pre-operative vascular population that demonstrated high inter-rater reliability in frailty assessment scores using the CFS between surgical and medical assistants and moderate correlation between the CFS scores and the Fried frailty index (Mirabelli, 2018). We identified that assessors with a medical background had the highest level of inter-rater reliability with the EFS frailty assessment tool compared to those from a nursing background. Several plausible explanations include consultants conducting a more comprehensive consultation and having greater experience in judging frailty based on clinical impression.

The statistically significant level of inter-rater reliability between the consultants and pre-assessment nurses is an important finding, suggesting that routine pre-operative assessment of frailty can be performed by pre-assessment nurses to an accuracy comparable with that of medically qualified clinicians. The CFS has moderate agreement in assessment of frailty status compared to the EFS. Clinically, the CFS is a more practical tool to use in a busy time pressured pre-assessment clinic environment than the ESF. It is less time intensive than the EFS, requiring no additional information or physical tests of a patient population who may be frail and living with marked functional disability, unlike some domains of the EFS.

However, the reliability of the CFS in identifying frailty in other patient groups cannot be extrapolated from this study. Vascular patients’ lifestyle risk factors and their disease process will often contribute to their frailty status. Visual cues of high-risk vascular surgical patients include smokers; abdominal obesity associated with type two diabetes; previous amputations. These stereotypes may allow for more accurate frailty assessment with the CFS than other surgical populations, for example colorectal cancer patients, who may be asymptomatic of their disease process.

The low number of severely frail patients recruited in our study is not reflective of the true prevalence of frailty in this surgical population as shown in the literature (Partridge et al., 2015). There are several different possible explanations for this. Firstly, the EFS and CFS may underreport severe frailty compared to the gold standards of frailty assessment in the research setting: the Comprehensive Geriatric Assessment (Parker, 2018). Secondly, there is a greater prevalence of frailty in the female population (Chan, 2019). Only 16.5% of our research population were female, reflecting that vascular disease is four to six times more common in men (Patel, 2016), and this may go some way to explaining our low numbers of more frail patients. Thirdly, it may also be that our vascular surgical team are adept at identifying severely frail patients based on clinical intuition and refer fewer to pre-assessment. This contradicts findings from other studies suggesting there is a lack of frailty knowledge across all disciplines within the hospital setting (Eamer, 2017); however, consistent involvement with anaesthetic high-risk pre-assessment clinics has increased surgeon awareness.

Although there were a low number of severely frail patients in our study, it is also important that there is consistency between all HCPs being able to reliably categorise patients across the frailty spectrum. This facilitates appropriate discussions with patients about surgical risk, better informs surgical decision making for non-frail and pre-frail patients as well as patients with frailty. Identifying non-frail patients allows for optimal resource utilisation, e.g. level one or two bed occupancy postoperatively. Identifying pre-frail as well as frail patients may allow for pre-habilitation to reduce frailty status (Stookey, 2020). This in turn may reduce surgical risk and improve patient outcomes (Hanna, 2019).

A possible limitation of our study is that it focused on assessment of frailty status in a homogenous vascular population in two hospitals in the north of England, potentially limiting more widespread geographical utility. In addition, study recruitment focused on the elective vascular surgical population, with the majority of patients undergoing repair of abdominal aortic aneurysm (AAA). Patients living with severe frailty would not usually even be considered for elective surgery for their AAA, which may have contributed significantly to the low number of frail patients recruited.

A further limitation pertains to the fact that we have not presented a truly reflective association between frailty status and perioperative outcomes. The sample size required for our primary analysis resulted in an insufficient number of patients recruited to make this analysis meaningful to present. Further work to identify how the frailty spectrum affects postoperative morbidity and mortality would facilitate more patient centred, tailored, approaches to healthcare including more individualised discussion about perioperative risk, consideration of postoperative levels of care required, discharge planning and work load management considering bed occupancy. We also acknowledge an imbalance in the number of patients recruited across the frailty groups limiting the utility of presentation of such an association.

## Conclusions

This observational study has demonstrated the feasibility and reliability of the CFS as an assessment tool for frailty in the pre-assessment setting in the vascular surgical population. The CFS is a more practical tool compared to the EFS for routine pre-operative frailty assessment. It can be easily incorporated into routine workflow of the pre-assessment of vascular patients with minimal barriers to implementation. This tool is non-cumbersome or time or resource intensive which makes it an attractive tool for this setting. Moreover, our study demonstrated that frailty assessment using the CFS assessment tool can be undertaken by HCPs without prior training making it an ideal tool to utilise in this setting. Unlike the EFS, use of the CFS is not limited for use in patients who do not speak English, or who are hearing or vision impaired or have other functional impairments such as mobility, ailments commonly encountered in the vascular population (Hilmer, 2009). In addition, our results suggest that frailty assessment ratings using the CFS tool are comparable to the reference tool, the EFS, and could easily be incorporated into routine clinical practice for assessment of frailty to promote perioperative risk stratification, risk modification and timely shared decision making to reduce the risk of adverse perioperative and postoperative outcomes.

## Data Availability

The datasets used and/or analysed during the current study are available from the corresponding author on reasonable request.

## Abbreviations

EFS: Edmonton Frailty Scale
CFS: Clinical Frailty Scale
STHNFT: South Tees Hospital NHS Foundation Trust
TYH: The York Hospital
HCP: Health care professional
IQR: Inter-quartile range
AAA: Abdominal aortic aneurysm

## References

[CR1] Amabili P, Wozolek A, Noirot I (2019). The Edmonton Frail Scale improves the prediction of 30-day mortality in elderly patients undergoing cardiac surgery: a prospective observational study. J Cardiothorac Vasc Anaesth..

[CR2] Amrock LG, Deiner S (2014). The implication of frailty on preoperative risk assessment. Curr Opin in Anaesthesiol..

[CR3] Centre for Perioperative Care and British Geriatric Society. Guideline for Perioperative Care for People Living with Frailty Undergoing Elective and Emergency Surgery. 2021. https://www.cpoc.org.uk/sites/cpoc/files/documents/2021-09/CPOC-BGS-Frailty-Guideline-2021.pdf (Acessed 15/12/21)

[CR4] Chan SP, Ip KY, Irwin MG (2019). Peri-operative optimisation of elderly and frail patients: a narrative review. Anaesthesia..

[CR5] Clegg A, Young J, Iliffe S, Rikkert MO, Rockwood K (2013). Frailty in elderly people. Lancet..

[CR6] Dasgupta M, Rolfson DB, Stolee P, Borrie MJ, Speechley M (2009). Frailty is associated with postoperative complications in older adults with medical problems. Arch Gerontol Geriatr..

[CR7] Dent E, Kowal P, Hoogendijk EO (2016). Frailty measurement in research and clinical practice: A review. Eur J Intern Med..

[CR8] Donald GW, Ghaffarian AA, Isaac F, Kraiss LW, Griffin CL, Smith BK, Sarfati MR, Beckstrom JL, Brooke BS (2018). Preoperative frailty assessment predicts loss of independence after vascular surgery. J Vasc Surg..

[CR9] Eamer G, Gibson JA, Gillis C (2017). Surgical frailty assessment: a missed opportunity. BMC Anesthesiology..

[CR10] Flaatten H, De Lange DW, Morandi A (2017). The impact of frailty on ICU and 30-day mortality and the level of care in very elderly patients (≥ 80 years). Intensive Care Med..

[CR11] Fried LP, Tangen CM, Walston J, Newman AB, Hirsch C, Gottdiener J, Seeman T, Tracy R, Kop WJ, Burke G, McBurnie MA (2001). Cardiovascular Health Study Collaborative Research Group. Frailty in older adults: evidence for a phenotype. J Gerontol Ser A Biol Sci Med Sci.

[CR12] Han B, Li Q, Chen X (2019). Effects of the frailty phenotype on post-operative complications in older surgical patients: a systematic review and meta-analysis. BMC Geriatrics..

[CR13] Hanna K, Ditillo M, Joseph B (2019). The role of frailty and prehabilitation in surgery. Curr Opin Crit Care..

[CR14] Health and social care informaion centre. Hospital episode statistics, admitted patient care activity. 2015. https://digital.nhs.uk/data-and-information/publications/statistical/hospital-admitted-patient-car-activity/hospital-episode-statistics-admitted-patient-care-england-2014-15. (Accessed 07/05/2019)

[CR15] Health and social care information centre. Hospital episode statistics, admitted patient care- England, 2006-2007: Main operations summary. 2006-2007. https://digital.nhs.uk/data-and-information/publications/statistical/hospital-admitted-patient-care-activity/hospital-episode-statistics-admitted-patient-care-england-2006-07 . (Accessed 07/05/2019)

[CR16] Hewitt J, Carter B, McCarthy K, Pearce L, Law J, Wilson FV, Tay HS, McCormack C, Stechman MJ, Moug SJ, Myint PK (2019). Frailty predicts mortality in all emergency surgical admissions regardless of age. An observational study. Age Ageing..

[CR17] Hewitt J, Moug SJ, Middleton M, Chakrabarti M, Stechman MJ (2015). McCarthy K; Older persons surgical outcomes collaboration. Prevalence of frailty and its association with mortality in general surgery. Am J Surg..

[CR18] Hilmer SN, Perera V, Mitchell S (2009). The assessment of frailty in older people in acute care. Australas J Ageing..

[CR19] Kramer HC, Thiemann S. How many subjects?: Statistical Power Analysis in Research. London: SAGE Publications; 1987.

[CR20] Landis JR, Koch GG (1977). The measurement of observer agreement for categorical data. Biometrics..

[CR21] McIsaac DI, Taljaard M, Bryson GL (2020). Frailty and longterm post-operative disability trajectories: a prospective multicentre cohort study. Br J Anaesth..

[CR22] Mirabelli LG, Cosker RM, Kraiss LW (2018). Rapid Methods for Routine Frailty Assessment during Vascular Surgery Clinic Visits. Ann Vasc Surg..

[CR23] O'Neill B, Hollingsworth AC, Batterham AM, Durrand JW, Danjoux GR (2016). Do first impressions count? Frailty judged by initial clinical impression predicts medium-term mortality in vascular surgical patients. Anaesthesia..

[CR24] Parker SG, McCue P, Phelps K (2018). What is Comprehensive Geriatric Assessment (CGA)? An umbrella review. Age Ageing.

[CR25] Partridge JS, Collingridge G, Gordon AL, Martin FC, Harari D, Dhesi JK (2014). Where are we in perioperative medicine for older surgical patients? A UK survey of geriatric medicine delivered services in surgery. Age Ageing..

[CR26] Partridge JS, Fuller M, Harari D, Taylor PR, Martin FC, Dhesi JK (2015). Frailty and poor functional status are common in arterial vascular surgical patients and affect postoperative outcomes. Int J Surg..

[CR27] Patel R, Sweeting MJ, Powell JT (2016). Greenhalgh RM; EVAR trial investigators. Endovascular versus open repair of abdominal aortic aneurysm in 15-years' follow-up of the UK endovascular aneurysm repair trial 1 (EVAR trial 1): a randomised controlled trial. Lancet.

[CR28] Pugh RJ, Thorpe CM, Subbe CP (2017). A critical age: can we reliably measure frailty in critical care?. Crit Care..

[CR29] Robinson TN, Wu DS, Pointer L, Dunn CL, Cleveland JC, Moss M (2013). Simple frailty score predicts postoperative complications across surgical specialties. Am J Surg..

[CR30] Rockwood K, Song X, MacKnight C, Bergman H, Hogan DB, McDowell I, Mitnitski A (2005). A global clinical measure of fitness and frailty in elderly people. Can Med Assoc J..

[CR31] Rockwood K, Theou O (2020). Using the clinical frailty scale in allocating scarce health care resources. Can Geriatr J.

[CR32] Rolfson DB, Majumdar SR, Tsuyuki RT, Tahir A, Rookwood K (2006). Validity and reliability of the Edmonton Frail Scale. Age Ageing..

[CR33] Schmucker AM, Hupert N, Mandl LA. The impact of frailty on short-term outcomes after elective hip and knee arthroplasty in older adults: a systematic review ‘published as epublication. Geriatr Orthop Surg Rehabil. 2019;10:1–12. 10.1177/2151459319835109. 10.1177/2151459319835109 PMC6503596 31105984

[CR34] Shears M, Takaoka A, Rochwerg B (2018). Assessing frailty in the intensive care unit: A reliability and validity study. J Crit Care..

[CR35] Song X, Mitnitski A, Rockwood K (2010). Prevalence and 10-year outcomes of frailty in older adults in relation to deficit accumulation. J Am Geriatr Soc J..

[CR36] Stookey AD, Katzel LI (2020). Home exercise interventions in frail older adults. Curr Geriatr Rep..

[CR37] Wang J, Zou Y, Zhao J, Schneider DB, Yang Y, Ma Y, Huang B, Yuan D (2018). The impact of frailty on outcomes of elderly patients after major vascular surgery: a systematic review and meta-analysis. Eur J Vasc Endovasc Surg..

